# Evaluating the impact of implementing public bicycle share programs on cycling: the International Bikeshare Impacts on Cycling and Collisions Study (IBICCS)

**DOI:** 10.1186/s12966-019-0871-9

**Published:** 2019-11-20

**Authors:** Kate Hosford, Meghan Winters, Lise Gauvin, Andi Camden, Anne-Sophie Dubé, Steven Marc Friedman, Daniel Fuller

**Affiliations:** 1 Faculty of Health Sciences, Simon Fraser University, 8888 University Drive, Burnaby, BC V5A 1S6 Canada; 2 Centre for Hip Health and Mobility, 2635 Laurel Street, Vancouver, BC V5Z 1M9 Canada; 3 Centre de recherche du Centre Hospitalier de l’Université de Montréal, 900, rue Saint-Denis, Pavillon R, Montréal, Quebec, H2X 0A9 Canada; 4 École de santé publique de l’Université de Montréal, 7101, Avenue du Parc, Montréal, Québec, H3N 1X9 Canada; 5 Division of Epidemiology, Dalla Lana School of Public Health, University of Toronto, Toronto, Ontario M5T 3M7 Canada; 6 Family & Community Medicine, University of Toronto, 500 University Avenue, Toronto, Ontario M5G 1V7 Canada; 7 School of Human Kinetics and Recreation, Memorial University of Newfoundland, Physical Education Building, St. John’s, Newfoundland A1C 5S7 Canada; 8 Department of Community Health and Humanities, Faculty of Medicine, Memorial University of Newfoundland, St. John’s, Newfoundland A1B 3V6 Canada

**Keywords:** Bicycle share, Bicycling, Evaluation, Natural experiment, Difference in differences

## Abstract

**Background:**

Despite rapid expansion of public bicycle share programs (PBSP), there are limited evaluations of the population-level impacts of these programs on cycling, leaving uncertainty as to whether these programs lead to net health gains at a population level or attract those that already cycle and are sufficiently physically active. Our objective was to determine whether the implementation of PBSPs increased population-level cycling in cities across the US and Canada.

**Methods:**

We conducted repeat cross-sectional surveys with 23,901 residents in cities with newly implemented PBSPs (Chicago, New York), existing PBSPs (Boston, Montreal, Toronto) and no PBSPs (Detroit, Philadelphia, Vancouver) at three time points (Fall 2012, 2013, 2014). We used a triple difference in differences analysis to assess whether there were increases in cycling over time amongst those living in closer proximity (< 500 m) to bicycle share docking stations in cities with newly implemented and existing PBSPs, relative to those in cities with no PBSPs.

**Results:**

Living in closer proximity to bicycle share predicted increases in cycling over time for those living in cities with newly implemented PBSPs at 2-year follow-up. No change was seen over time for those living in closer proximity to bicycle share in cities with existing PBSPs relative to those in cities with no PBSP.

**Conclusion:**

These findings indicate that PBSPs are associated with increases in population-level cycling for those who live near to a docking station in the second year of program implementation.

## Background

Public bicycle share programs (PBSP) make bicycles available for rent throughout a city. As of 2014, there were over 855 public bicycle share programs operating in cities around the world [1]. PBSPs are implemented for a variety of reasons, including to increase cycling levels, facilitate the first and last miles of public transportation trips, and reduce traffic congestion amongst other objectives [2, 3]. These programs are used for both transportation and leisure trips but are primarily intended to facilitate short transportation trips (< 30 min). Typically, PBSPs users are required to pay an additional fee for any single trip that exceeds the designated time limit (usually 30 min). Potential pathways through which these programs may lead to increases in population-level cycling are by increasing access to bicycles for those who do not own their own bicycle, increasing the convenience of bicycling, and normalizing bicycling as a form of transportation [2, 4].

Despite rapid expansion of PBSPs, there are limited evaluations of the population-level impacts of these programs on cycling, leaving uncertainty as to whether these programs lead to net increases in cycling at a population level or attract those that already cycle and are sufficiently physically active [5]. The evidence base is further limited to case studies conducted in one city or studies that do not have pre-intervention measures or control groups [5, 6].

Mixed results are reported from two pre-post evaluations of PBSPs in Canada. In Montreal, a study showed a greater likelihood of cycling in the second season of program operation for those who lived near a bicycle share docking station compared to those who did not [7]. A similar study in Vancouver in 2017 also found initial increases in the likelihood of cycling for those who lived and worked near a bicycle share docking station, but this increase was not sustained into the second season of program operation [8]. Differing results between the two cities could be explained in part by the much larger scale of Montreal’s PBSP compared to Vancouver’s in the early phase of program implementation [8].

Evidence across multiple cities and contexts is needed to better understand the impacts of PBSPs on health [5, 6]. It is possible that weak associations or null findings reported in single city studies are due partly to limited power to detect intervention effects or account for contextual factors, such as a slower than anticipated rollout of a bike share program or wet and rainy weather during follow-up data collection [8]. Multi-city studies can address this gap, by collecting data across a variety of contexts and increasing the power to detect intervention effects.

In the current study, we used data from 8 major North American cities to estimate the impact of PBSPs on population-level cycling from 2012 to 2014 as part of the International Bikeshare Impacts on Cycling and Collisions Study (IBICCS) [6]. Cities either had recently implemented a PBSP in 2013, had an existing PBSP in place, or had no PBSP. We hypothesized that the greatest increase in the likelihood of cycling over time would be observed in cities with a recently implemented PBSP, followed by cities with existing PBSPs. Since cycling is typically more common among men in North America, [9, 10] we also examined whether or not associations differed between men and women.

## Methods

### IBICCS

IBICCS is a quasi-experimental non-equivalent groups study designed to evaluate the health impacts of PBSPs. The study protocol is published elsewhere [6]. In brief, we sampled residents (aged 18+ years) in cities that either had a newly implemented PBSP in 2013 (Chicago, New York), an existing PBSP (Boston, Montreal, Toronto), or no PBSP during the study period (Detroit, Philadelphia, Vancouver) (*n* = 23,901). We used online repeated cross-sectional surveys at three time points (Fall 2012, 2013, and 2014) to collect data on cycling behaviour, sociodemographic characteristics, self-reported health, and residential location. Fall data collection is consistent with Household Travel Behaviour Surveys and is timed to ensure participants were engaged in regular bicycle commuting behaviour. The Centre de Recherche du Centre Hospitalier de l’Université de Montréal and Toronto Public Health granted ethics approval for all IBICCS study procedures and survey respondents provided informed consent.

### Study cities

The IBICCS sample included data from residents in 8 cities, with varying population sizes and land areas (Table 1). The proportion of the working population that commuted to work by bicycle was low across all cities, ranging from 0.4% in Detroit to 4.4% in Vancouver. Of the five cities that had a PBSP, Toronto had the smallest program with 78 stations and Montreal had the largest with 444 stations as of Fall 2014.

**Table 1:** City population, bicycling mode share, and PSBP information for the 8 cities where respondents were sampled in IBICCS

	City							
	Newly implemented PBSP		Existing PBSP			Control		
	Chicago	New York	Boston	Montreal	Toronto	Detroit	Philadelphia	Vancouver
Population^a^	2,695,598	8,175,133	617,594	1,649,519	2,615,060	713,777	1,526,006	603,502
Area (km^2^)	589.6	783.8	125.0	365.1	630.2	359.4	347.3	115.0
Population density (per km^2^)	4571.9	10,430.1	4940.8	4517.6	4149.5	1986.0	4393.9	5249.2
Proportion who commute to work by bicycle^b^	1.4%	1.0%	1.9%	3.1%	2.2%	0.4%	2.0%	4.4%
Public bicycle share program (PBSP)	Divvy Bikes	City Bike	Blue Bikes	BIXI Montréal	Bike Share Toronto	–	–	–
PBSP implementation	2013	2013	2011	2009	2011	–	–	–
# of docking stations, 2014	300	332	140	444	78	–	–	–

^a^Chicago, New York, Boston, Detroit, and Philadelphia based on 2010 Census [11]. Montreal, Toronto, and Vancouver based on 2011 Canadian Census [12]

^b^Chicago, New York, Boston, Detroit, and Philadelphia based on 2010–2014 American Community Survey 5-year estimates [13]. Montreal, Toronto, and Vancouver based on 2011 National Household survey [14]

### Measures

The outcome was a binary measure of self-reported cycling for any purpose (transportation or leisure) for at least 10 min in the past week or not. We derived the binary measure from the long version of the validated International Physical Activity Questionnaire (IPAQ) transport physical activity module [15]. Respondents were asked to report the average number of minutes they bicycled per week for transportation or leisure in the past month.

The primary independent variables used in the difference in differences analysis were time (baseline, year 1, year 2), exposure to the PBSP service area (living nearer, living further away from), and city type (newly implemented, already existing, none). For respondents in cities with PBSPs, we assigned exposure to the PBSP service area based on whether they had one or more bicycle share docking stations within a 500 m road network buffer of their home. We selected a distance of 500 m because it represents a reasonable distance to walk to a docking station and has been used in previous studies to define service areas [7, 8, 16]. For respondents residing in control cities with no PBSP, we assigned exposure in a similar way based on a hypothetical PBSP. Since 2015, all three control cities implemented a PBSP (Philadelphia in 2015, Vancouver in 2016, and Detroit in 2017). Therefore, we used the docking station locations 1 year into each program’s launch to assign exposure to the PBSP service area in control cities. In Detroit, we used 2.5 km buffers to assign exposure instead of 500 m buffers because Detroit’s bike share program is small relative to the size of the city (*n* = 43 stations) and only 48 participants were assigned as exposed when using 500 m buffers, which was not sufficient for our modelling approach. There were three city types: newly implemented, already existing, and no PBSP. Newly implemented cities had a PBSP implemented mid-way through the study period, already existing PBSP cities had a bicycle share program in place for the duration of the study period, and control cities did not have a PBSP during the study period.

We identified covariates a priori based on socio-demographic characteristics and environmental factors that have been used in previous evaluations of PBSPs [7, 8] and that could influence cycling, including gender (men, women), age (18–24, 25–34, 35–44, 45–54, 55+), education attainment (high school or less, college or university), employment status (employed, student, retired, unemployed), having children in the household (none, one child, more than one child), self-reported health (poor/fair/good, very good/excellent), and Walk Score [17] at participants home postal or ZIP code (car-dependent: < 50, somewhat walkable: 50–69, very walkable: 70–89, walker’s paradise: 90–100). Ethnicity was originally collected as White/Caucasian, Black/African/African-American, Hispanic/Latino/Spanish, Native American/American Indian, Arab (Middle East, North Africa), Indian/Pakistani, Other specified, or refused. We recoded the ethnicity categories as White, Black, Hispanic/Latino, Asian, Mixed/Other, and refused to ensure sufficient sample size for statistical analysis.

### Analysis

We used census data to apply post-stratification weights on age and gender to adjust the sample to better represent the general population. We trimmed survey weights at 0.2 and 10 [18].

To assess if PBSP had an impact on cycling, we used a triple difference in differences analysis [19, 20]. The goal of this analysis is to determine whether cycling increased more over time for those residing in and around the areas where PBSP stations are located (or would be implemented) in newly implemented and existing PBSP cities relative to those in control cities (i.e., counterfactual scenario). Logistic regression was used to estimate unadjusted and adjusted odds ratios (ORs) and 95% confidence intervals (CIs). This method models the outcome (odds of cycling in the past week) in a model that includes time (baseline, 1-year follow-up, 2-year follow-up), exposure (nearer vs. further away), city type (control, newly implemented PBSP, or existing PBSP), interaction terms between time, exposure, and city type, and covariates. The three-way interaction term between time, exposure, and city type estimates the effect of the PBSP on the odds of cycling over time for those exposed to PBSP in cities with newly implemented and existing PBSPs relative to those exposed in control cities and is therefore the primary effect of interest in this analysis. We fit individual models for each covariate listed above, followed by an adjusted model containing all variables. The fully adjusted model had a lower Akaike Information Criterion (AIC) compared to the unadjusted model, indicating a better model fit. We also conducted a sensitivity analysis excluding Detroit as a control city because it had a lower proportion of people who bicycle to work and lower population density then the other cities and fewer respondents were assigned as exposed compared to the other control cities. Effect measure modification by gender was examined using stratified logistic regression models. All analyses were conducted in R version 3.5.1.

## Results

### Sample characteristics

The pooled sample included 23,901 respondents with 7829 respondents in 2012, 7979 in 2013, and 8093 in 2014. Cooperation rates, which is the proportion of eligible respondents who agree to participate, were 12, 9, and 4%, respectively. Of the 23,901 respondents, 878 were excluded because they did not provide a home postal code or zip code (*n* = 284) or were missing socio-demographic data (*n* = 594). Our final sample included 23,023 respondents (96.3% of initial sample), resulting in a weighted population of 22,164.

Table 4 in Appendix 1 shows the socio-demographic characteristics of the study sample and compares them to the population using the National Household Survey and the American Community Survey. The weighted sample is representative of the population in terms of age and gender due to post-stratification weighting, but there was notable underrepresentation of people of color and those with lower education levels and incomes. The selection bias in the study sample is similar across cities with newly implemented PBSPs, existing PBSPs, and no PBSPs, which suggests that comparisons between cities meets the assumptions of difference in differences despite the sample not being completely representative of the population.

Weighted socio-demographic characteristics and proportions reporting cycling for at least 10 min in the previous week by year are shown in Table 2. In the overall sample, the proportion of respondents that reported past-week cycling increased from 18.1% in 2012 to 24.8% in 2013 and 24.5% in 2014. Past-week cycling is broken down by exposure to PBSP, city type, and gender in Table 5 in Appendix 2. Across exposure levels and city type, the proportion that cycled increased over time, with the largest increases occurring between 2012 and 2013 (Table 5, Appendix 2). In terms of gender, disproportionately fewer women reported cycling in the previous week compared to men at baseline (14.0% compared to 22.7% for men), however, the proportion reporting cycling over time increased for both women and men (Table 5, Appendix 2). In the overall sample, the proportion reporting use of PBSP bicycles also increased over time, from 9.0% of the sample reporting use of a PBSP bicycle in 2012 to 14.3% in 2014 (Table 2).

**Table 2:** Weighted characteristics of survey respondents in the IBICCS sample with complete socio-demographic data, 2012–2014 (*n* = 23,023, n weighted = 22,164)

	Fall 2012 (Baseline)		Fall 2013 (1-year Follow-Up)		Fall 2014 (2-year Follow-up)	
	weighted *n* (%)		weighted *n* (%)		weighted *n* (%)	
Total	6782	(30.6)	7508	(33.9)	7875	(35.5)
Cycling in the past week (yes)	1226.4	(18.1)	1863.5	(24.8)	1929.7	(24.5)
Bike share use (yes)^a^	613.1	(9.0)	953.9	(12.7)	1127.4	(14.3)
Exposed to PBSP (yes)	2909.2	(42.9)	2049.6	(27.3)	3111.0	(39.5)
City type						
Control (no PBSP)	2481.2	(36.6)	2812.2	(37.5)	2945.3	(37.4)
Newly implemented PBSP	1625.3	(24.0)	1879.7	(25.0)	1965.5	(25.0)
Existing PBSP	2675.5	(39.4)	2815.6	(37.5)	2963.7	(37.6)
Gender (men)	3164.1	(46.7)	3569.1	(47.5)	3761.4	(47.8)
Age (years)						
18–24	1025.6	(15.1)	1002.2	(13.3)	1051.8	(13.4)
25–34	1780.1	(26.2)	1772.5	(23.6)	1850.4	(23.5)
35–44	1307.2	(19.3)	1292.7	(17.2)	1337.8	(17.0)
45–54	1231.2	(18.2)	1242.1	(16.5)	1289.9	(16.4)
55+	1437.9	(21.2)	2198.0	(29.3)	2344.6	(29.8)
Education attainment						
High school or less	546.3	(8.1)	1092.4	(14.6)	1004.9	(12.8)
College or University	6235.7	(91.9)	6415.2	(85.4)	6869.6	(87.2)
Employment status						
Employed	5015.2	(73.9)	5081.9	(67.7)	5225.3	(66.4)
Student	718.3	(10.6)	637.6	(8.5)	712.0	(9.0)
Retired	560.1	(8.3)	961.7	(12.8)	1160.2	(14.7)
Unemployed	488.4	(7.2)	826.4	(11.0)	777.1	(9.9)
Ethnicity						
White	5043.9	(74.4)	5502.7	(73.3)	5450.6	(69.2)
Black	261.9	(3.9)	560.2	(7.5)	729.8	(9.3)
Hispanic/Latino	182.5	(2.7)	269.9	(3.6)	329.4	(4.2)
Asian	878.2	(12.9)	766.8	(10.2)	905.4	(11.5)
Mixed/Other	267.0	(3.9)	274.4	(3.7)	289.6	(3.7)
Refused	148.4	(2.2)	133.6	(1.8)	169.5	(2.2)
Children in household						
None	5594.7	(82.5)	5750.6	(76.6)	6163.7	(78.3)
One child	677.5	(10.0)	950.8	(12.7)	933.5	(11.9)
More than one child	509.8	(7.5)	806.2	(10.7)	777.2	(9.9)
Self-reported health						
Poor/Fair/Good	2451.0	(36.1)	3020.9	(40.2)	3158.8	(40.1)
Very good/Excellent	4331.0	(63.9)	4486.7	(59.8)	4715.7	(59.9)
Walk Score of home neighborhood						
Car dependent (< 50)	864.7	(12.8)	1548.5	(20.6)	813.0	(10.3)
Somewhat Walkable (50–69)	696.1	(10.3)	1118.8	(14.9)	863.7	(11.0)
Very Walkable (70–89)	1664.5	(24.5)	1962.7	(26.1)	2180.0	(27.7)
Walker’s Paradise (90–100)	3556.6	(52.4)	2877.6	(38.3)	4017.9	(51.0)

^a^Bike share use in any city (including a PBSP outside of one’s home city)

Approximately two-thirds of the sample were from a city that implemented PBSP during the study period (*n* = 5471, 24.7%) or a city that had an existing PBSP (*n* = 8455, 38.1%). Exposure to PBSP did vary by year, with fewer respondents exposed in 2013 (27.3% of total sample) compared to 2012 (42.9%) and 2014 (39.5%) (Table 2). A greater proportion of respondents who were exposed reported having used a PBSP bicycle in their home city (~ 25%) compared to unexposed respondents (~ 10–15%) (Table 6 in Appendix 3).

### Impact of PBSP on cycling

Weighted logistic regression models examining the odds of cycling over time for exposed and unexposed respondents in cities with PBSP and control cities are shown in Table 3. We focus the discussion of results on the three-way interaction term (time x exposure x city type) because this estimates the difference in the average change in cycling for exposed residents in cities with PBSPs compared to exposed residents in control cities.

**Table 3:** Results of weighted difference in differences logistic regression models estimating associations between past-week cycling, time, exposure to PBSP, city type, and their interactions, in the overall sample and stratified by gender, 2012–2014

	Overall (weighted *n* = 22,164)		Men (weighted *n* = 10,494)		Women (weighted *n* = 11,670)	
	Unadjusted OR (95% CI)*	Adjusted** OR (95% CI)	Unadjusted OR (95% CI)	Adjusted* OR (95% CI)	Unadjusted OR (95% CI)	Adjusted* OR (95% CI)
Time*Exposure*City type						
2013*exposed*control (ref)	1.00	1.00	1.00	1.00	1.00	1.00
2013*exposed*newly implemented	**2.14 (1.11, 4.12)**	1.85 (0.95, 3.61)	1.68 (0.66, 4.29)	1.48 (0.56, 3.88)	**2.61 (1.08, 6.32)**	2.42 (0.98, 5.94)
2013*exposed*existing	0.91 (0.51, 1.62)	0.93 (0.51, 1.68)	0.98 (0.43, 2.22)	1.00 (0.43, 2.34)	0.77 (0.34, 1.71)	0.81 (0.36, 1.85)
2014*exposed*control (ref)	1.00	1.00	1.00	1.00	1.00	1.00
2014*exposed*newly implemented	**2.08 (1.14, 3.77)**	**1.84 (1.003, 3.39)**	**2.31 (1.01, 5.32)**	1.94 (0.82, 4.57)	1.67 (0.73, 3.78)	1.72 (0.75, 3.93)
2014*exposed*existing	1.00 (0.59, 1.70)	1.02 (0.59, 1.76)	1.34 (0.63, 2.86)	1.39 (0.63, 3.03)	0.68 (0.33, 1.43)	0.70 (0.33, 1.48)

Bold is the confidence interval with a lower value of 1.00

^a^OR = Odds ratio; 95% CI = 95% Confidence interval

^b^Adjusted models control for age, gender, education attainment, employment status, ethnicity, having children in the household, self-rated health, and Walk Score in residential neighbourhood

In the unadjusted model, there was over two-fold greater odds of past-week cycling for those living near a docking station in cities with newly implemented PBSPs at the first follow-up (OR: 2.14, 95% CI: 1.11, 4.12) and second follow-up (OR: 2.08, 95% CI: 1.14, 3.77), relative to those in cities with no PBSP. The effect size was attenuated when adjusting for socio-demographic factors and Walk Score in residential neighbourhood but remained significant at the second follow-up point (OR: 1.84, 95% CI: 1.003, 3.39). There was no differential change in cycling over time for those living near a docking station in cities that had an existing PBSP, relative to those in cities with no PBSP. The results of the sensitivity analysis excluding Detroit (Table 7 in Appendix 4) showed that the magnitude of association for the three-way interaction term was reduced but the direction of the association did not change (OR: 1.77, 95% CI: 0.92, 3.38).

Stratifying the model by gender showed that there may be some nuanced shifts in the gender composition of cyclists in cities with newly implemented PBSPs. In the unadjusted model, the odds of past-week cycling were two and half times greater for women living nearer to a docking station in cities with newly implemented PBSPs at the first follow-up (OR: 2.61, 95% CI: 1.08, 6.32) compared to women in cities with no PBSP. This association was also in the positive direction in the adjusted model, but the confidence interval crossed 1 (OR: 2.42, 95% CI: 0.98, 5.94). This trend was not sustained into the second year for women living near to a docking station. Conversely, the odds of past-week cycling for men living nearer to a docking station at the first follow-up point was not different relative to men in cities with no PBSP, but increased at the second follow-up. The odds were two times greater for men living nearer to a docking station in the unadjusted model (OR: 2.31, 95% CI: 1.01, 5.32) relative to the reference group, but when adjusting for covariates, the confidence interval crossed 1 (OR: 1.94, 95% CI: 0.82, 4.57).

**Fig. 1:**
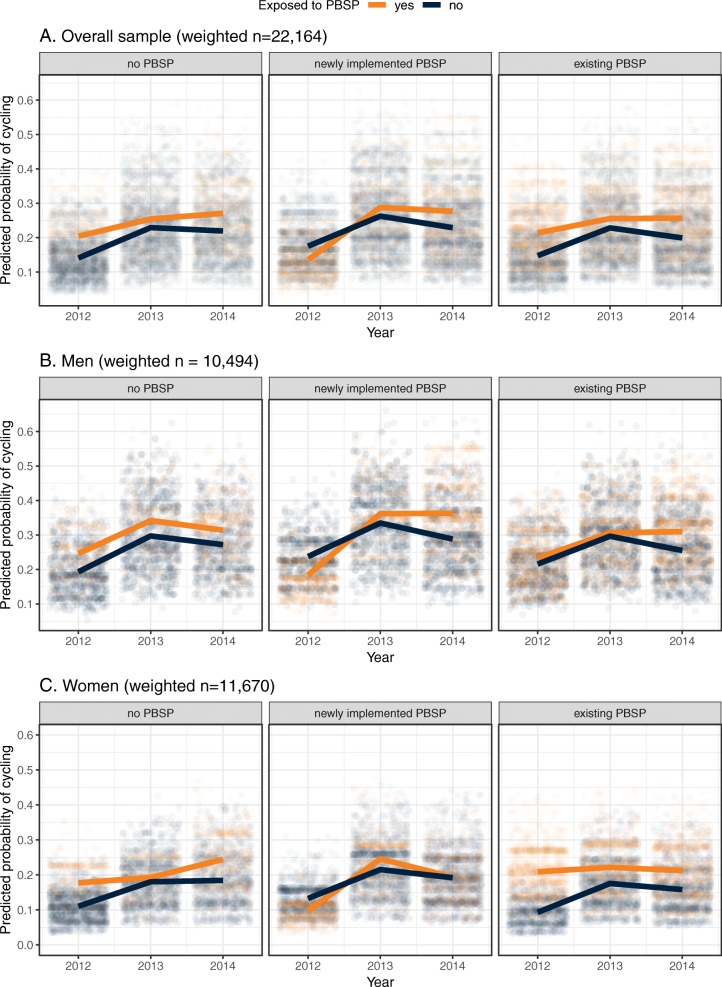
Fully adjusted predicted probability of cycling by exposure to PBSP in cities with no PBSP, newly implemented PBSPs, and existing PBSPs, in the overall sample and stratified by gender, 2012–2014

Figure 1 shows the model-based estimate of the predicted probability of cycling for each individual (points) and on average (lines) from 2012 to 2014 across city types, for exposed and unexposed respondents in the overall sample and stratified by gender. The triple interaction term in the difference in differences analysis controls for baseline differences between groups and for changes in cycling for those living further away from the PBSP (not exposed) and those living in cities with no PBSP (control cities) (i.e., impacts on cycling that can be attributed to other causes). From this figure, we can see that the mean predicted probability of cycling increases more over time for those who are exposed living in cities with newly implemented PBSPs relative to those who are not exposed, as well as compared to those who are living in cities with no PBSP (Panel 1a). We can also see that women living near to a docking station in cities with newly implemented PBSPs appear to increase their cycling more at the first follow-up point (Panel 1c) and men at the second follow-up (Panel 1b), relative to respondents who are not exposed and respondents living in cities with no PBSP.

## Discussion

This large-scale multi-city study examined whether PBSP increased population-level cycling in cities with newly implemented and existing PBSPs. Overall, we found that PBSPs were associated with an increase in self-reported cycling for those living near to a docking station in cities with newly implemented PBSPs at 2-year follow-up relative to those in cities no PBSP. On average, residents living within 500 m of a bicycle share docking station had 1.8 times the odds of reporting past-week cycling at 2-year follow up, compared to residents living in comparable areas in cities without PBSPs. Examining the effects for women and men separately showed that PBSPs may be associated with a greater likelihood of cycling for women living near to a docking station in cities with newly implemented PBSBs 1-year into program implementation and for men 2-years into program implementation.

The observed increase in cycling could be from both the use of PBSP bicycles and personal bicycles. In these samples, the proportion that had ever used a PBSP bicycle increased from 9.0% in 2012 to 14.3% in 2014. PBSP can make cycling more convenient by allowing for one-way trips by bicycle, eliminating the concern of bicycle theft, facilitating the first and last miles of transportation trips, and by providing access to bicycles for those who do not own their own bicycle [16, 21, 22]. Past surveys with PBSP members report that members overall indicated their frequency of cycling increased since joining a PBSP and that a substantial proportion do not own a personal bicycle [23, 24]. PBSP use can also be a gateway to riding a personal bicycle more often or contribute to normalizing the image of cycling in a city [4]. An observational study in London showed that PBSP users were less likely to wear a helmet, high visibility clothing, or sport clothing compared to cyclists using personal bicycles, which could encourage non-cyclists to start cycling by increasing the number and diversity of cyclists [4].

We hypothesized that the greatest increases in cycling would be observed in cities with newly implemented PBSPs, but we also anticipated that there would be increases in cycling in cities with existing PBSPs relative to cities with no PBSPs. It may be that the gains in population cycling level off after the first few years of a PBSP’s implementation. For instance, ridership of BIXI bicycles in Montreal increased from when it was first implemented in 2009 to 2011 but stabilized between 2012 and 2014 [25], which could explain in part why we did not observe an increase in cycling in cities with existing PBSPs. In addition, the parent company for Montreal and Toronto’s public bike share program, Public Bike System Company, faced financial problems during 2013 and filed for bankruptcy in early 2014, which could have contributed to negative perceptions of bike sharing, and by extent use of the public bike share programs during the study period.

An important contribution of the current analysis is that we investigated the impact of PBSP on cycling for men and women separately. In countries with low cycling rates, there remains a significant gender gap in cycling [9, 10, 26, 27]. In Canada and the US, men are estimated to cycle 2–3 times as much as women, in terms of probability of commuting to work and number of trips [9, 10]. Most surveys of PBSP members in North America reveal that there is also a gender gap in PBSP membership, with men being more likely to have a bike share membership compared to women [2, 28, 29]. However, some evidence suggests that the gender gap in public bicycle sharing may be less than the gap in cycling in general [24]. In this study the longer term effect of a new PBSP on the gender gap in overall cycling is unclear. In cities with newly implemented bike share programs, we observed an initial increase in cycling for women living near to a docking station in the first year of program implementation relative to women in cities with no PBSP, but this did not sustain into the second year. Longer term studies are needed to confirm whether any subsequent gains are observed for women.

### Strengths and limitations

This study surveyed almost 24,000 people in 8 cities across 3 time-points to estimate the impact of a population-wide intervention on cycling. We focus on population-level cycling as an outcome. This is a common goal of bike share programs [2, 3], albeit the policy context and original program goals motivating the implementation of a specific program are rarely transparent, and often not clear from documents on city or program operator’s websites [2]. By using the difference in differences approach, we controlled for the counterfactual change in cycling (i.e., to what extent would the likelihood of cycling change if PBSPs had not been implemented). The triple difference in differences analysis added another layer by controlling for changes in cycling between populations residing nearer to a PBSP station (or where a PBSP station would likely be located) and populations that did not. This approach offers an improvement over methods that do not include pre-post measurements or control groups; making an important contribution to understanding the public health implications of implementing public bicycle share programs in urban areas.

This study has limitations common to natural experiment studies including bias from residual confounding, selection bias, exposure assignment, the unpredictable nature of real-world interventions, and challenges of multi-country studies [6]. There could be residual confounding in the analyses from unobserved factors that had different effects across exposure groups or cities. For example, we did not control for changes to cycling infrastructure because publicly available data for cycling infrastructure are not available for most cities. If increases in infrastructure were more substantial in areas of the newly implemented PBSPs, this may relate to increases in cycling. Additionally, our surveys were matched for time of year but changes in weather during follow-up data collection could have impacted the proportion of respondents that reported past-week cycling. A second limitation of this study is selection bias. We weighted our sample by age and gender to better represent the underlying population, however, there was notable underrepresentation of people of colour, and those with lower education levels and incomes, especially in some cities (Table 4 in Appendix 1). Moreover, a key assumption of the difference in differences method is that there are no substantial changes in the sociodemographic structure of the study sample overall, or in the exposed and unexposed groups. Though we used the same recruitment methodology across years, there were fewer respondents exposed in 2013 (27.3% of total sample) compared to 2012 (42.9%) and 2014 (39.5%). In terms of exposure assignment, we considered respondents who live within 500 m of PBSP stations to be exposed because past studies have found that those who live near to docking stations are more likely to use PBSP and increase their cycling [7, 8, 16]. However, PBSPs are also used by those who live further than 500 m from bike share docking stations, such a misclassification would likely bias results towards the null. Lastly, implementation of PBSPs across cities did not occur as expected. Control cities initially selected for the IBICCS study were Chicago (comparison for New York, Montreal, and Toronto), Detroit (for Boston), and Philadelphia (for Vancouver). Vancouver’s PBSP did not launch during the study period and Chicago, a planned control city, launched their PBSP in 2013. Consequently, we had to shift Vancouver to a control city and Chicago to an intervention city. These real-world conditions limit the exchangeability assumptions between cities, but the method is still an improvement over past research as we include cities with no PBSP as controls [3].

Finally, the measure of cycling that we used for our outcome was a binary measure of whether the respondent self-reported cycling in the past week. Future studies may wish to include a measure for frequency and duration of cycling to determine whether PSBPs increase the volume of cycling at a population level.

## Conclusions

Using a large-scale quasi-experimental multi-city study, we observed that PBSPs were associated with increases in population-level cycling for those who live near to a docking station in the second year of program implementation in cities with newly implemented PBSPs. This finding suggests that PBSPs can contribute to increases in cycling at a population-level.

## Data Availability

The datasets generated and analyzed during the current study are not publicly available because participants were assured their data would remain confidential and would not be shared.

## Appendix 1

### Sample representativeness

**Table 4:** Comparison of selected socio-demographic characteristics of IBICCS study participants with comparison datasets to assess sample representativeness (*n* weighted = 22,164).

		Newly Implemented PBSP city				Existing PBSP city						No PBSP city					
		Chicago		New York		Boston		Montreal		Toronto		Detroit		Philadelphia		Vancouver	
		IBICCS	ACS^a^	IBICCS	ACS	IBICCS	ACS	IBICCS	census^b^	IBICCS	census	IBICCS	ACS	IBICCS	ACS	IBICCS	census
# of census tracts in sampled area		345		335		145		333		91		73		107		59	
		(%)		(%)		(%)		(%)		(%)		(%)		(%)		(%)	
Education	High school education or less	8.0	32.1	7.8	25.5	6.6	27.4	15.6	36.2	11.6	32.8	15.5	54.4	16.0	41.2	14.1	29.9
	College or University	92.0	67.9	92.2	74.5	93.4	72.6	84.4	63.8	88.4	67.2	84.5	45.6	84.0	58.8	85.9	70.1
Employment	Employed	77.8	65.3	73.3	64.6	69.6	62.2	62.8	58.1	69.9	65.0	63.7	38.8	66.1	54.1	70.1	67.7
	Unemployed	4.4	6.5	4.6	5.4	3.2	5.4	5.1	6.4	3.6	5.5	3.8	12.6	4.6	6.7	2.7	4.9
	Not in labor force	17.8	28.3	22.1	30.0	27.2	32.4	32.2	35.4	26.5	29.5	32.5	48.5	29.3	39.3	27.2	27.5
Household Income	< 50 k	31.8	44.4	29.5	37.1	35.7	44.0	45.5	60.0	31.5	45.8	39.8	78.4	42.7	55.0	31.1	48.3
	> = 50 k	68.2	55.6	70.5	62.9	64.3	56.0	54.5	40.0	68.4	54.2	60.2	21.6	57.3	45.0	68.8	51.7
Ethnicity	White	71.8	44.5	68.0	53.1	75.6	58.1	84.7	70.4	70.9	64.3	78.5	15.1	69.2	49.0	58.4	64.4
	Black	11.1	15.6	8.7	14.6	4.7	13.6	2.4	6.9	2.3	5.4	11.6	67.9	15.3	28.9	0.8	1.2
	Hispanic/Latino	6.8	29.0	7.7	17.0	3.7	12.1	1.9	4.4	1.6	2.4	1.5	12.8	4.2	8.2	1.2	1.9
	Asian	6.9	8.6	11.7	12.5	11.6	12.1	4.4	11.5	14.5	24.2	3.7	1.9	7.8	10.9	31.0	30.2
	Mixed/Other	1.9	2.3	2.3	2.8	2.5	4.1	4.5	5.9	7.5	3.2	3.1	2.2	2.4	3.0	5.5	2.0
	Refused	1.4	–	1.7	–	1.8	–	2.0	–	3.3	–	1.5	–	1.2	–	3.2	–

^a^ACS = American Community Survey; Source: 2010–2014 American Community Survey 5-year estimates [9]

^b^Source: 2011 National Household survey [10]

^c^Refused responses for Income were removed from the IBICCS study sample to allow for comparisons with American Community Survey data and the National Household Survey

## Appendix 2

### Past-week cycling by exposure to PBSP, city type, and gender

**Table 5:** Weighted proportion of survey respondents in the IBICCS sample who cycled for at least 10 min in the past week by exposure status, city type, and gender, 2012–2014 (*n* weighted = 5020)

	Fall 2012 (Baseline)		Fall 2013 (1-year Follow-Up)		Fall 2014 (2-year Follow-up)	
	weighted % reporting cycling		weighted % reporting cycling		weighted % reporting cycling	
Total reporting cycling in the past week for at least 10 min	1226.4	18.1%	1863.5	24.8%	1929.7	24.5%
Exposed to PBSP						
Yes		20.9%		27.2%		27.6%
No		15.9%		23.9%		22.5%
City type						
Control (no PBSP)		17.3%		23.6%		24.7%
Newly implemented PBSP		16.3%		26.1%		25.3%
Existing PBSP		19.9%		25.2%		23.8%
Gender						
Women		14.0%		19.4%		19.6%
Men		22.7%		30.8%		29.8%

## Appendix 3

### Bicycle share use by exposure to PBSP, city type, and gender

**Table 6:** Weighted proportion of survey respondents in the IBICCS sample who reported having used a public bike share bicycle in the city in which they live at least once, by exposure to PBSP, 2012–2014

	Fall 2012 (Baseline)	Fall 2013 (1-year Follow-Up)	Fall 2014 (2-year Follow-up)
	weighted % reporting bike share use^a^	weighted % reporting bike share use	weighted % reporting bike share use
Newly implemented PBSP city		16.5%	18.9%
Exposed to PBSP	–	24.3%	26.9%
Not exposed to PBSP	–	14.4%	15.1%
Existing PBSP city	18.5%	18.2%	19.3%
Exposed to PBSP	25.7%	25.8%	25.8%
Not exposed to PBSP	9.1%	13.1%	12.9%

^a^Based on the survey question, “Have you ever used one of these public bicycle share bicycles in the city in which you live?”

## Appendix 4

### Sensitivity analysis excluding Detroit as a control city in logistic regression models

**Table 7:** Results of weighted difference in differences logistic regression models estimating associations between past-week cycling, time, exposure to PBSP, city type, and their interactions when Detroit is excluded as a control city, in the overall sample and stratified by gender, 2012–2014

	Overall (weighted *n* = 19,390)		Men (weighted *n* = 9204)		Women (weighted n = 10,186)	
	Unadjusted OR (95% CI)^a^	Adjusted^b^ OR (95% CI)	Unadjusted OR (95% CI)	Adjusted* OR (95% CI)	Unadjusted OR (95% CI)	Adjusted* OR (95% CI)
Time*Exposure*City type						
2013*exposed*control (ref)	1.00	1.00	1.00	1.00	1.00	1.00
2013*exposed*newly implemented	1.90 (0.94, 3.83)	1.61 (0.79, 3.30)	1.38 (0.51, 3.71)	1.19 (0.43, 3.29)	2.59 (0.99, 6.74)	2.43 (0.92, 6.42)
2013*exposed*existing	0.81 (0.43, 1.52)	0.82 (0.43, 1.56)	0.80 (0.33, 1.94)	0.81 (0.33, 2.03)	0.76 (0.31, 1.84)	0.81 (0.33, 2.01)
2014*exposed*control (ref)	1.00	1.00	1.00	1.00	1.00	1.00
2014*exposed*newly implemented	1.77 (0.92, 3.38)	1.55 (0.80, 3.02)	1.94 (0.79, 4.77)	1.59 (0.63, 4.03)	1.50 (0.61, 3.70)	1.54 (0.62, 3.82)
2014*exposed*existing	0.85 (0.47, 1.53)	0.85 (0.46, 1.57)	1.13 (0.49, 2.58)	1.13 (0.48, 2.68)	0.61 (0.27, 1.41)	0.62 (0.27, 1.44)

^a^OR = Odds ratio; 95% CI = 95% Confidence interval

^b^Adjusted models control for age, gender, education attainment, employment status, ethnicity, having children in the household, self-rated health, and Walk Score in residential neighbourhood
